# Editorial Comment: Female urethroplasty: contemporary thinking

**DOI:** 10.1590/S1677-5538.IBJU.2020.01.06

**Published:** 2020-01-13

**Authors:** C West, A Lawrence, Luciano A. Favorito

**Affiliations:** 1 Eastern Health, Box Hill, VIC, Australia; 2 Auckland Hospital, Auckland, New Zealand; 1 Unidade de Pesquisa Urogenital - Universidade Estadual do Rio de Janeiro - Uerj, Rio de Janeiro, RJ, Brasil;; 2 Serviço de Urologia, Hospital Federal da Lagoa, Rio de Janeiro, RJ, Brasil

## COMMENT

In this interesting review West and Lawrence show important points about a major and poorly studied topic: the female urethral stricture. In the review the authors show the management options and observed that multiple open urethroplasty techniques are described with various grafts and flaps, with good medium-term success. Minimally invasive techniques remain well-employed but have poor long-term success, with increased failure with multiple attempts at treatment and concluded that the use of vaginal flaps and buccal mucosal graft present interesting success rates and urethral dilation should be avoided due to disappointing long-term results.

An important topic to be discussed is what is the best option for surgical reconstruction of female urethral strictures with buccal mucosa graft? Ventral or dorsal approach? What is the best choice? Both dorsal and ventral approaches are acceptable options for the surgical reconstruction of female urethral strictures (1). Dr. Nayak in an interesting paper show that ventral-inlay buccal mucosal graft urethroplasty is a simple and safe method of urethroplasty in women with good results (2). In other interesting paper Dr Manasa shows that early functional results after dorsal onlay vaginal graft urethroplasty are good without any negative impact on continence or sexual functions. The dorsal buccal mucosa grafts have gained popularity, because they maintain intact the ventro-lateral urethral supporting structures, important for continence (3). In an interesting paper by Gomez et al, the authors show a success rate of the dorsal graft of 86% of the patients in a long follow-up with low morbidity (3). In this unusual disease, the use of buccal mucosa graft for treatment seems to be the best choice.

## References

[1] 1. Nayak P, Mandal S, Das M. Ventral-inlay buccal mucosal graft urethroplasty for female urethral stricture. Indian J Urol. 2019;35:273-277. 10.4103/iju.IJU_57_19 PMC6792411 31619865

[2] 2. Manasa T, Khattar N, Tripathi M, Varshney A, Goel H, Sood R. Dorsal onlay graft urethroplasty for female urethral stricture improves sexual function: Short-term results of a prospective study using vaginal graft. Indian J Urol. 2019;35:267-72. 10.4103/iju.IJU_134_19 PMC6792406 31619864

[3] 3. Gomez RG, Segura FJ, Saavedra A, Campos RA. Female urethral reconstruction: dorsal buccal mucosa graft onlay. World J Urol. 2019 Sep 21. [Epub ahead of print]. 10.1007/s00345-019-02958-6 31542825

